# Planning a clinical research study

**DOI:** 10.4103/0019-5413.30520

**Published:** 2007

**Authors:** Simon Chan, Anders Jönsson, Mohit Bhandari

**Affiliations:** Division of Orthopedic Surgery, McMaster University, Hamilton, Ontario, Canada; * Clinical Research Group, Association Internationale pour l' Ostéosynthèse, Dynamique (AIOD)

In planning any research protocol, we should consider two questions: 1. Is there a real need for the trial? 2. Is the study design and methodology robust? We focus on the second issue-study validity.

A randomized controlled trial (RCT) is the most valid of the clinical research designs. It is a prospective study where allocation to the treatment groups is random. Recently, RCTs have become widespread in the medical literature. In 1998, more than 12000 RCTs were being published each year, more than double the annual publication rate of just a decade previously.^1^ This growth can be traced to the growing acceptance of RCTs as the most reliable experimental design for investigating therapeutic interventions.^2^ Although preferred, RCTs are just one of many research designs [Table 1].

**Table 1:** Study designs^6^

Type of study	Description	Pros	Cons
Case series	Subjects with interesting outcomes are presented	-Cheap	-Selection bias
		-Fast	-No control group
Cross-sectional	Simultaneous assessment of exposure and outcome in a group	-Useful in studying prevalence outcomes	-No temporal relationship between exposures and
Case-control	Subjects selected based on outcome and then exposure is assessed	-Overcomes temporal delays-May only require small sample size	-Selection and recall bias
			-Temporal relationship may not be clear
Cohort	Subjects with and without exposure are followed	-Feasible when randomization of exposure is not possible	-Critically depends on follow-up
			-Classification and measurement accuracy
RCT	Exposure is randomly determined	-Least susceptible to bias	-Feasibility
			-Generalizability

While outside factors such as cost or time may influence the choice of design, the most suitable research design is dictated by the research question being asked.^3^ For example, it would be unethical to randomize patients to an exposure suspected as being harmful. A cohort study would be an appropriate and ethical design to answer such a question. Nonetheless, for questions of therapy, RCTs have moved to the top of what is known as the therapeutic hierarchy [Table 2]. The validity of the evidence is highest for a single, large randomized trial.^4^ Randomization limits bias and controls for unknown prognostic variables.^5^ Careful deliberation of some simple questions can help to ensure a valid, robust RCT [Table 3].

**Table 2:** The therapeutic hierarchy^32^

Single large randomized controlled trial
Systematic review of several small randomized controlled trials
Single small randomized controlled trial
Systematic review of several cohort studies
Single cohort study
Systematic review of several case-control studies
Single case-control study
Systematic review of several cross-sectional studies
Single cross-sectional study
Case series

**Table 3:** How to assess a randomized trial

**Will the results be valid?**
How will potential sources of bias be avoided?
What is the justification for the hypothesis underlying the power calculations?
**Will the results be applicable?**
Has sufficient account been taken within the study design of the issues of generalizability and representativeness?
Is the trial population reflective of the target population so that the results will have meaning?
Have the outcome measures been well chosen and adequately defined?

## WILL THE RESULTS BE VALID?

This first section of this paper deals with **internal validity.**

## HOW WILL POTENTIAL SOURCES OF BIAS BE AVOIDED?

Bias is “a systematic tendency to produce an outcome that differs from the underlying truth”.^6^ Bias in clinical trials falls into four categories: selection bias, performance bias, detection bias and attrition bias [Table 4].^7^

**Table 4:** Forms of bias

Selection bias	biased allocation to comparison groups
Performance bias	unequal provision of care apart from treatment
Detection bias	biased assessment of outcome
Attrition bias	biased occurrence and handling of deviations from protocol and loss to follow-up

### Selection bias

The goal when enrolling patients is to create comparison groups that are similar with respect to all known or unknown confounding factors. This is accomplished by randomizing patients. Reviews comparing randomized with observational studies have found that a lack of randomization can lead to both underestimation and overestimation of the treatment effect.^8^ The process of randomization depends on two procedures: generation of an allocation sequence and allocation concealment [Table 5].

**Table 5:** Treatment allocation

Generation of allocation sequence
Definition: The creation of an allocation sequence based on a random process.
Considered adequate are randomizations by dice; tables of random numbers; computer-generated sequences; etc
Considered inadequate are randomizations by date of birth; chart number, day of admission, alternating; etc
**Allocation concealment**
Definition: The process of ensuring that no one knows of the group assignment prior to randomization.
Considered adequate are serially-numbered, opaque, sealed envelopes; sequentially numbered containers; pharmacy-controlled; central randomization (investigators phone/fax or go online to obtain next group assignment); etc
Considered inadequate are alternating, use of containers or envelopes that can be compromised; etc

### Randomization

Fundamental to RCTs is the random allocation of patients to comparison groups.^9^ Nonrandom methods of allocation subvert the whole purpose of an RCT. Some methods are described as “pseudorandomization”.^10^ Examples include allocating patients by chart number, date of presentation or by alternating assignment. There is the risk of introducing bias into your study. As an example, in some populations the day of week on which a child is born is not a completely random event.^11^ There is also the risk of compromising allocation concealment if your allocation sequence is predictable.

While there are complex methods of generating an adequate allocation sequence, the most elegant and simple designs are underused. These include a table of random numbers or a computer-generated sequence.

Groups are more likely to be balanced as the sample size increases when using a random number generator. For example, in a sample size of 20 patients, investigators should expect that roughly 10% of the sequences generated via simple randomization would yield a ratio imbalance of three to seven or worse.^12^ Manual methods of randomization such as coin-tossing or dice are technically correct, but are less preferable since they allow the implementer to sabotage the randomization. For example, when flipping a coin, a series of heads or a series of tails may occur. An investigator may be tempted to alter the result of a coin toss in order to rectify what they perceive to be a nonrandom sequence, when in fact their actions serve to do just the opposite. Another disadvantage of these manual methods of randomization is that they leave no paper trail and so cannot be checked at a later date.

### Concealment of allocation

A proper allocation concealment scheme keeps investigators and patients unaware of upcoming assignments. In an ideal world, allocation concealment would be unnecessary and patients would enter into the trials groups to which they were originally assigned. It is important to realize however, that the process of randomization often frustrates clinical inclinations. In cases of poor allocation concealment (for example, posting of the allocation sequence), knowledge of upcoming assignments could lead to the exclusion of patients the care provider felt were unsuited for a particular treatment group.

Recognize also that the forces being placed upon healthcare providers may be stronger than the forces pushing for adherence to an RCT protocol. In these cases, even good attempts at allocation concealment may be subverted, as was the case in one study where residents held envelopes up to bright light to decipher upcoming assignments to avoid hassling their attendings with the more involved treatment late at night.^13^ The importance of allocation concealment in protecting against bias has been shown in a study that showed greater heterogeneity in trials with improperly concealed allocation.^14^

Development of a robust method of allocation concealment requires thought and effort. In addition to the demands of day-to-day medicine which frequently trump the desire to maintain good research methodology, one must also contend with human nature and the natural inclination of some to decipher the concealed allocation for curiosity's sake alone.

When designing a trial, use of additional elements to ensure that your concealment is tamper-proof is advised [Table 6].^15^

**Table 6:** Concealment

Good	Better
Sequentially numbered, opaque, sealed envelopes	Use of pressure-sensitive or carbon paper to transfer information Material within envelope (foil, cardboard) to ensure opacity
Sequentially numbered containers	All containers are tamper-proof Containers are identical in appearance and weight
Pharmacy-controlled	Indication that investigator developed or validated randomization scheme used by pharmacy
Central randomization	Description of the mechanism for contact, Ensure enrolment into study before assignment

### Performance bias and detection bias

Performance bias arises when the treatment assignment is known to patients or caregivers, and detection bias arises when outcome assessors or data analysts are similarly aware. They will be considered together since the solution for both is the same. Blinding is the process of ensuring that such parties are kept unaware of whether patients have been assigned to a treatment or a control group. Without blinding securely in place, an RCT is vulnerable to bias from a number of sources [Table 7].^16^

**Table 7:** Blinding

Not blinded	Danger
Participant	May have biased psychological or physical response to intervention
	Less likely to comply with trial regimen
	More likely to seek adjunct intervention
	More likely to leave trial without providing outcome data
Caregiver	May transfer attitudes and clinical inclinations to patients
	More likely to administer co-interventions
	More likely to adjust dose
	More likely to differentially withdraw participants
	More likely to differentially encourage or discourage participants to continue trial
Outcome assessor	More likely to have biases affect assessment of outcome
Data analyst	More likely to have biases affect analysis of data

The importance of blinding to preventing personal bias from clouding judgment is especially important when assessing subjective outcomes. One study has shown that nonblinded assessors were more likely to see the benefit of an intervention than blinded assessors.^17^ Blinding of certain parties may be impossible in some trials. As an example, it may not be possible to blind caregivers or outcome assessors in surgical trials. The absence of blinding does not preclude the ability to create a methodologically strong RCT. As an example, use of objective outcome measures or assessment by a third party not involved with the RCT are viable methods to avoid bias when blinding of outcome assessors is not possible. Sometimes the administration of a noneffective treatment can have a positive effect on outcomes because the patient believes it will work. This phenomenon is known as the placebo effect. Aside from helping to compensate for the placebo effect, use of a placebo in the control group is an important aspect of blinding. Patients and physicians would quickly discern allocation assignments if the treatment between comparison groups was readily observed to be different. Whenever possible, an inert, but otherwise identical placebo should be used.

### Attrition bias

Throughout the course of a trial, there will be participants who deviate from the study protocol or those who drop out and refuse any further participation. This population of patients may differ in a relevant and systematic way from the patients who have adhered to the trial protocol. As an example, patients may have dropped out and become unavailable for further follow-up due to acute exacerbations of their illnesses.^18^ Likewise, it would not be surprising if those patients who suffered the most serious side-effects were those who chose to deviate from the study protocol. For these reasons, the analysis should include all randomized patients, not just those who adhered to the treatment protocol. In addition, all patients should be analyzed according to the groups to which they were originally allocated, regardless of what treatment they actually received. This type of analysis is known as intention-to-treat and guards against the introduction of attrition bias.^19^ However, exclusion from the analysis is sometimes unpreventable. This occurs if some participants become lost to follow-up before outcomes can be recorded. In such circumstances, it is important to report explicitly the number of subjects excluded and to discuss the possibility of attrition bias in the written report. Strategies to maximize patient follow-up are presented in Table 8.^19^ Tips for avoiding bias in a clinical trial are presented in Table 9.

**Table 8:** Approaches to maximizing participant follow-up

Hire a person to manage and encourage follow-up
Hire personnel to call participants or visit participants at their homes or places of work, if participants are not returning for follow-up
Exclude before randomization those likely to be unwilling to return
Exclude before randomization those likely to move
Obtain contact information to prompt participants to return for follow-up and facilitate location of participant if they do not return
Obtain an identification number, such as a national healthcare number
Establish follow-up venues suited to participants rather than to investigators
Streamline trial procedures to move participants quickly through a follow-up visit
Keep data collection instrument short
Provide excellent and free medical care
Provide monetary subsidies

**Table 9:** Tips for avoiding bias

Keep randomization simple
Spend the time and effort to design a tamper-proof method of allocation concealment.
Leave an audit trail
Blind as many of the following as possible: study enroller, participant, caregiver, outcome assessor, data analyst
Make sure the placebo is well designed
Use intention-to-treat analysis

## SAMPLE SIZE, HYPOTHESIS-TESTING AND STUDY POWER

The goal of any RCT design is to use the smallest sample size necessary to attain a prespecified level of *power* to detect an effect of interest.^20^ Power is just one factor to consider when determining sample size. It is not the intent of this article to show how sample size calculations are derived. The focus will instead be on the four key factors that must be considered in all sample size formulae [Table 10].^21^

**Table 10:** Key factors in sample size formula

Level of Significance	Tells us how likely it is that an observed difference is due to chance when no true difference exists
Power of test	Tells us how likely we are to detect an effect
Variance	A measure of the variability of any characteristic. Varies from sample to sample.
Effect size	The magnitude of the difference between comparison groups for any characteristic

When testing a hypothesis, we risk making two types of fundamental errors [Table 11].^22^,^24^ Type I errors occur when we conclude that the treatment had an effect, when it in fact did not. The probability of making a Type I error is known as the significance level of the test and is denoted as α. Type II errors occur when we conclude that the treatment had no effect, when in fact it did. The probability of a Type II error is denoted by β. Power is 1- β and it represents the probability of avoiding a false-negative conclusion.

**Table 11:** Errors in hypothesis testing truth

		Difference	No difference
Results of study	Difference	Correct	Type I error/False-positive
	No difference	Type II error/False-negative	Correct

Typically, α is set at 0.5 and β is set at 0.20, giving rise to a power of 0.80. Stated in words, this means that we're willing to accept a 5% chance of making a false-positive conclusion and that we have an 80% chance of detecting a difference between comparison groups, if a true difference exists.

Variance and effect size have opposite effects on sample size. As the effect size increases, the necessary sample size decreases. The larger the effect size, the more easily it would be detected, so it makes sense intuitively that fewer subjects (less information) would be needed.^20^ As the variance increases, the necessary sample size increases as well. This can be illustrated by imagining a population where the variance was zero, which is to say that each member of the population was identical. In this case, the sample size could be very small and still be a good representation of the population.

As the level of significance (β) and power (1-β) of the test are often set at β =0.05 and 1-β =0.80 respectively, our influence on the sample size comes from our estimations of variance and effect size. Variance will depend upon the population under investigation and the reliability of the tool being used to measure outcomes. Estimations of both variance and effect size can come from historical data and from examination of similar populations. While much subjective judgment is involved, it is important to temper optimism when making these estimations. Overestimation of effect size will result in too few subjects and an RCT that is under-powered.^23^ It may be worthwhile to undertake a pilot study to ensure that your estimations of variance and effect size are realistic. This may also be helpful in helping predict the anticipated rates of noncompliance and loss to follow-up. Again, failure to account for these factors will lead to a decrease in sample size. The resulting study would then lack the power to impact clinical practice and research in a meaningful way.^24^,^25^

## WILL THE RESULTS BE APPLICABLE?

The second half of this article deals with the issues of applicability and clinical utility. A study is said to have good **external validity** if its results will generalize to the larger population.

### Has sufficient account been taken within the study design of the issues of generalizability and representativeness?

The trial setting is often a source of concern regarding generalizability. Physicians in primary care often wrestle with the applicability of RCT results obtained in tertiary and secondary centers.^26^ Often, primary care patients suffer numerous comorbidities that would have been exclusion criteria in the very studies that examine the efficacy of the therapies relevant to them.^27^

The differences between countries with regards to their demographics and healthcare systems can also affect external validity. Racial differences can affect the natural history or susceptibility to a disease.^28^ Regional differences in the diagnosis and treatment of the same disease may be strikingly different. This can lead to differences in the use of adjuvant, nontrial treatments. For example, in an international RCT of aspirin and heparin for acute ischemic stroke, glycerol was used in 50% of the 1473 patients in Italy versus 3% elsewhere.^29^ In addition to adjuvant therapies, consideration should also be given to the generalizability of the entire treatment protocol. In order to have broad applicability, the RCT protocol should diagnose and manage patients pretrial and posttrial in a manner that mirrors actual clinical practice.^30^

### Is the trial population reflective of the target population so that the results will have meaning?

To maintain external validity, it is important that the sample population be representative of the whole. For many reasons, this may not be the case. To begin with, recruiting for trials is often undertaken by specialists in tertiary care centers. From the outset, this group of patients will differ from those patients being managed in the community by primary care physicians. Often, this threat to validity can never fully be eliminated since a certain proportion of the population never presents at a location or time that is conducive to entry into a trial. However, attempts to rectify it can be made by sampling before other selection pressures impose themselves. A trial's eligibility criteria are then applied to arrive at an even more selective group. Attempts to remove confounding factors and diagnoses can lead to stringent eligibility criteria and very high exclusion rates. An average exclusion rate of 73% was found in a review of 41 US National Institutes of Health RCTs.^31^ Strict eligibility criteria create a sample that is again less representative of the population, which limits external validity. This is compounded by the fact that participating clinicians may apply additional selection criteria beyond that of the eligibility criteria. While usually done with altruistic intentions (clinicians seek to enroll those they feel will do well in the trial.), this practice further deteriorates the representativeness of the sample population.

### Have the outcome measures been well chosen and adequately defined?

As noted previously in this paper, we typically accept a 5% probability of obtaining a false-positive when testing a hypothesis. For this reason, it is important to limit the number of investigated outcomes. The more the outcomes evaluated, the greater the chance of obtaining a false-positive result.

The applicability of an RCT depends on the clinical relevance of the measured outcomes. There has been a shift towards the use of simple, clinically relevant outcomes and away from surrogate outcomes.^32^ Surrogate outcomes are often misleading. Observational studies may show correlation between a surrogate outcome and a relevant clinical outcome and a treatment may show a positive effect on that same surrogate outcome, yet the treatment may still be ineffective harmful. Antiarrythmic drugs used to be prescribed for postmyocardial infarction to reduce ECG abnormalities (the surrogate outcome). This ceased becoming the standard of care when RCTs showed increased mortality (clinically relevant outcome) due to this treatment.^33^

The use of inappropriate scales or composite scores is also harmful to external validity. Unvalidated scales have been found to be more likely to show significant treatment effects than validated scales.^34^ In addition, the clinical relevance of an apparent treatment effect (i.e. a 5-point mean reduction on a 50-point outcome scale made up of various signs and symptoms) is impossible to determine.^30^

Trials can gain statistical power by combining multiple outcomes to form a composite outcome. Unfortunately, composite outcomes can hurt the applicability of an RCT result. The treatment may affect each individual outcome in different ways. The results of an RCT reporting a composite outcome may not be applicable to a patient who is particularly predisposed to developing one of the specific outcomes. Another danger is when outcomes of varying severities are combined. Less serious outcomes often occur more frequently. In this case, the least clinically significant outcome would have an inordinate impact on treatment effects.

Careful consideration should also be given to the patient and disease process. Patients typically prioritize quality of life issues more than clinicians, who tend to focus on the physical aspects of a disease. Since the final goal is to uncover therapies that improve things for patients, it makes sense to adopt patient-centered outcomes.

The RCTs investigating chronic diseases have often suffered from inadequate duration of follow-up. Clinicians treat these patients over months and years and the results of a RCT with follow-up measured in weeks are of limited applicability.^35^

## SUMMARY

RCTs provide the most reliable data when investigating questions of therapy. For this reason, they play a central role in helping clinicians make evidence-based decisions. However, it requires much planning and thought to design a robust RCT that possesses good internal and external validity. Care should be taken to use proper methodology to avoid bias. An adequate sample size should be obtained so as to avoid an underpowered study. Efforts should be made to make the sample as representative of the population as possible. Simple, clinically relevant outcomes should be used.

Even the perfectly designed and executed RCT would be useless if those reading the report are not aware of its quality. Issues of quality of reporting are intertwined with issues of methodological quality. The use of quality of reporting as an indicator of methodological rigor is problematic because the two do not always correlate.^36^ A well-conducted but poorly reported study may not receive proper credit, while a biased but well reported study may wield undue influence. Guidelines on the reporting of clinical trials have been developed to combat this problem.^2^ As a final point, this author would also like to encourage investigators to think longitudinally. Try and stay one step ahead of your participants and anticipate any problems or concerns that may arise [Table 12]. The process of conceiving, developing and organizing an RCT can be long and arduous, but if done properly, can serve to advance clinical medicine.

**Table 12:** Summary points

Avoid bias by blinding and randomizing
Think carefully about allocation concealment
Use intention-to-treat analysis
Ensure your sample size gives your study enough power
Make your study setting and sample population as representative as possible
Use simple, clinically relevant outcomes
Be clear and explicit when reporting RCT methodology
Think longitudinally
